# Strategic white matter injury associated with long‐term information processing speed deficits in mild traumatic brain injury

**DOI:** 10.1002/hbm.25135

**Published:** 2020-07-13

**Authors:** Lijun Bai, Guanghui Bai, Shan Wang, Xuefei Yang, Shuoqiu Gan, Xiaoyan Jia, Bo Yin, Zhihan Yan

**Affiliations:** ^1^ Department of Radiology The Second Affiliated Hospital and Yuying Children's Hospital of Wenzhou Medical University Wenzhou China; ^2^ The Key Laboratory of Biomedical Information Engineering, Ministry of Education, Department of Biomedical Engineering School of Life Science and Technology, Xi' an Jiaotong University Xi'an China; ^3^ Department of Neurosurgery the Second Affiliated Hospital and Yuying Children's Hospital of Wenzhou Medical University Wenzhou China

**Keywords:** DTI, information processing speed, mild traumatic brain injury, prognosis, serum inflammation cytokine

## Abstract

Deficits in information processing speed (IPS) are among the earliest and most prominent cognitive manifestations in mild traumatic brain injury (mTBI). We investigated the impact of white matter fiber location on IPS outcome in an individual basis assessment. A total of 112 acute mild TBI with all CT negative underwent brain DTI and blood sampling for inflammation cytokines within 7 days postinjury and 72 age‐ and sex matched healthy controls with same assessments were enrolled. IPS outcome was assessed by the trail making test at 6–12 month postinjury in mild TBI. Fractional anisotropy (FA) features were extracted using a novel lesion‐load analytical strategy to capture spatially heterogeneous white matter injuries and minimize implicit assumptions of uniform injury across diverse clinical presentations. Acute mild TBI exhibited a general pattern of increased and decreased FA in specific white matter tracts. The power of acute FA measures to identify patients developing IPS deficits with 92% accuracy and further improved to 96% accuracy by adding inflammation cytokines. The classifiers predicted individual's IPS and working memory ratings (*r* = .74 and .80, respectively, *p* < .001). The thalamo‐cortical circuits and commissural tracts projecting or connecting frontal regions became important predictors. This prognostic model was also verified by an independent replicate sample. Our findings highlighted damage to frontal interhemispheric and thalamic projection fiber tracts harboring frontal‐subcortical neuronal circuits as a predictor for processing speed performance in mild TBI.

## Graphical abstract

Combination of inflammation cytokine and fibers projecting to frontal cortex as predictors can identify long‐term information processing speed deficits in mild TBI patients with negative CT.

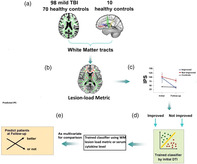

## 1 INTRODUCTION

Traumatic brain injuries (TBI) is a public health challenge of vast, but insufficiently recognized, proportions. Mild traumatic brain injury (TBI) accounts for 80–90% of all cases of TBI worldwide (Levin & Diaz‐Arrastia, 2015; Thornhill et al., 2000). Approximately 30% patients will harbor persistent cognitive deficits that contribute to life changing sequelae (Max, Mackenzie, & Rice, 1991; Sharp, Scott, & Leech, 2014). Of which, reduced information processing speed (IPS) are pervasive, precede clinical diagnosis, and form the core of TBI‐associated cognitive disabilities (Draper & Ponsford, 2008). Potential therapeutic strategies (i.e., catecholaminergic drugs) are available and improve IPS. Therefore, identification of theranostics biomarkers for mild TBI with developing IPS deficits is essential that can guide the use of treatment that enhances individual cognition.

The clinical assessment of this persisting cognitive deficit is challenging, especially when no gross abnormalities indicative of the cognitive or functional loss are detected on routine diagnostic imaging (e.g., structural MRI and CT). Only 4% of mild TBI have relevant or suspected pathological findings (Smith, 2012), leading to the larger proportion of patients receiving no medical attentions. It is suggested that abnormalities of selective swelling and disconnection of white matter axons following trauma can be better detected by diffusion tensor imaging (DTI) than by conventional imaging (Niogi & Mukherjee, 2010; Shenton et al., 2012). However, heterogeneous and inconsistent conclusions have been drawn regarding to the direction of tissue water diffusivity abnormalities, including increased (Henry et al., 2011; Ling et al., 2012; Mayer et al., 2010), decreased during the initial diagnose (acute or semi‐acute stage) (Messe et al., 2012; Miles et al., 2008). Specially, this claim of traumatic axonal injury has been challenged by recent study reporting null changes in mild TBI (Ilvesmaki et al., 2014). Besides variability due to clinical factors, the analytical approach used for detecting white matter abnormalities may attribute to discrepancies in mild TBI DTI studies. In general, previous studies assumed that clinically heterogeneous patients have a homogenous (i.e., high degree of spatial overlap) pattern of white matter abnormalities. To address this question, we adopted a novel approach by measuring diffusion abnormalities through a metric similar to lesion‐load (White, Schmidt, & Karatekin, 2009). Specifically, clusters of abnormally high or low anisotropic diffusion were determined on a voxel‐wise basis and then summed to represent total burden of distributed pathology. Such bi‐directional changes in fractional anisotropy (FA) are also detected after early injury in the rodent controlled cortical impact (CCI) model (Harris, Verley, Gutman, & Sutton, 2016), as well as following subacute of mild TBI (Ling et al., 2012).

TBI effects on white matter as well as white matter effects on cognition are region specific (Kinnunen et al., 2011). It is reported that the structure of the fornix is related to the efficiency of working memory and the anterior corona radiata associated with executive function following mild TBI (Kinnunen et al., 2011). Chronic decreased FA in the anterior forceps is associated with persistent verbal letter fluency impairment after mild head injury (Croall et al., 2014). However, these studies primarily focus on the relation between microstructural changes and cognition within either acute or chronic stage, potential of very early injury of specific white matter tracts in identifying the long‐term cognitive outcome is still unclear. Among this, processing speed presents as the most sensitive cognitive domains to mild TBI (Karr, Areshenkoff, Duggan, & Garcia‐Barrera, 2014). It is supposed that the physiological effects of mild TBI interact with the aging process and exacerbate cognitive impairments (Henry, Tremblay, & De Beaumont, 2017). Models of cognitive aging proposes the processing speed as the fundamental cognitive process to support higher cognitive functions (i.e., working memory) and drive general declines (Salthouse, 2009). Information processing speed (IPS) depends on large‐scale, long‐distance neural network operations that are supported by myelinated neuronal axonal fibers (Bartzokis et al., 2010; Waxman & Bennett, 1972). Therefore, the existence of specific white matter abnormalities maybe an important predictor to classify subtype of mild TBI patients with differential IPS outcome. Additionally, our recent study also demonstrates that acute serum inflammation cytokine levels (i.e., chemokine ligand 2, CCL2) can predict long‐term IPS profiles in participants with mild TBI (Sun et al., 2019). Ultimately, combination of both DTI metrics and inflammation cytokine at the very early acute phase can provide better predictor to help clinicians rule out long‐term cognitive impairment in acute mild TBI.

In the past few years, our research group has followed a relatively large sample of civilian mild TBI and acquired various measurements of their neuropsychological function, brain imaging and blood serum (Niu et al., 2019; Wang et al., 2019; Xu et al., 2018). We have identified several cognitive and brain abnormalities in the group level, but we never combined these into an integrated biological signature that could be used at the individual level. The emerging field of machine learning provide a way to identify an integrated biological signature for unbiased diagnostic purposes. Here, a longitudinal study combining MRI‐DTI and inflammation cytokine with multivariate pattern analysis aimed to test: (a) whether diffusion metrics combined with inflammation cytokine during early acute mild TBI contained sufficient information to identify patients with IPS deficits over 6–12 months postinjury; (b) whether the predicted model based on IPS training can be adopted to provide predictions of working memory in individuals, considering mediation effects of IPS observed in healthy and neurological related changes in working memory; (c) whether this predict model can be independently replicate in a new cohort of patients using identical experimental protocols to enhance its general applicability. We hypothesized that deficits in processing speed would be related to white matter lesions at strategic locations.

## 2 METHODS

### 2.1 Participants

The study involved 112 mild TBI patients and 72 healthy controls from two independent cohorts (as original and replicated samples) (Table 1). The original cohort was enrolled from March 2014 to Oct. 2015 and replicated sample from April 2016 to Dec 2018 respectively, and all of datasets for these two cohort were collected from the same center and using the same scanner. Inclusion and exclusion criteria were maintained for both samples and reported in our previous studies (Niu et al., 2019). Screening for mild TBI was based on the World Health Organization's Collaborating Centre for Neurotrauma Task Force (Holm, Cassidy, Carroll, & Borg, 2005). All the subjects gave written, informed consent in person approved by the Local Institutional Review Board and conducted in accordance with the Declaration of Helsinki.

**TABLE 1:** Demographic and behavioral statistics for patients with mild TBI and healthy controls (Mean ± *SD*)

Patients characteristic	Original sample			Replicate sample		
	Patients	Controls	*P* value	Patients	Controls	*P* value
Age	35.3 (14.8)	36.5 (13.6)^a^	.77 (−0.14)	37.0 (11.2)	37.3 (8.9)^a^	.95 (−0.03)
Gender	*35/25*	25/15^a^	.84 (0.08)	20/18	18/12^a^	.36 (−0.24)
Educational level	*8.1 (4.1)*	10.8 (4.9)^a^	.12 (−0.38)	8.7 (4.2)	10.4 (3.6)^a^	.21 (−0.43)
Neuropsychological testing						
TMT‐A (at initial)	*58.7 (44.5)*	28.3 (8.0)	<.001 (1.55)	58.7 (26.8)	30.0 (10.8)	<.001 (1.41)
TMT‐A (at follow‐up)	51.2 (39.3)	27.6 (3.5)	<.001 (1.48)	59.3 (38.9)	32.0 (12.3)	<.001 (0.96)
BDS (at initial)	*3.9 (1.6)*	5.1 (1.9)	<.001 (−1.48)	3.2 (1.8)	5.2 (2.0)	<.005 (−0.76)
BDS (at follow‐up)	*3.7 (1.4)*	4.9 (1.3)	<.005 (−0.84)	3.9 (1.7)	5.1 (2.7)	<.005 (−0.51)
Serum cytokines						
*IL‐6*	*1.3 (2.4)*	0.9 (0.3)	<.001 (1.84)	1.2 (1.9)	0.8 (0.5)	<.001 (0.96)
*CCL2*	*259.7 (116.7)*	*212.3 (50.9)*	<.001 (0.86)	276.6 (140.5)	211.1 (79.1)	<.001 (1.61)
*IL‐1*β	*2.6 (0.8)*	2.2 (0.9)	<.001 (0.99)	3.1 (1.0)	2.3 (1.2)	<.001(1.19)

Abbreviations: BDS, backward digit span; CCL2, chemokine ligand 2; IL1, interleukin‐1; IL6, interleukin‐6; TMT A, trail‐making test part A.

### 2.2 Clinical assessment and outcome

Consistent clinical and cognitive assessments were maintained for both samples, and were conducted at both initial (acute phase, within 7 days postinjury) and follow‐up (6–12 month postinjury). All of patients in the present study were free of litigation to avoid any bias on the testing performance. The neuropsychological tests mainly included the information processing speed (IPS rated by Trail Making Test A) (Arnett & Labovitz, 1995) and working memory (by Backward Digit Span from the WAIS‐III) (Harman‐Smith, Mathias, Bowden, Rosenfeld, & Bigler, 2013). For the cognitive information processing speed measured by the Trail A, the split criteria were based on the norms adjusted by both age and education level (Tombaugh, 2004).

### 2.3 Serum biomarkers

Acute mild TBI patients and matched healthy controls were collected the serum samples, and the details were reported in our current study (Sun et al., 2019). The 9‐plex panel of serum cytokines included the interleukin (IL)‐1β, IL‐4, IL‐6, IL‐8, IL‐10, IL‐12, chemokine ligand 2 (CCL2), interferon‐γ (IFN‐γ), and tumor necrosis factor‐α (TNF‐α). In the present study, we found that serum levels of IL‐1β, IL‐6, and CCL2 were acutely elevated in mild TBI patients relative to controls (Table 1). Thus, these three serum biomarkers were selected as predictor features.

### 2.4 MRI imaging

The protocol for scanning included a noncontrast CT scan for acute head injury. The MRI (3 T GE 750) protocol for each subject (mild TBI patients and controls) included the high‐resolution T1‐weighted 3D MPRAGE sequence (TE = 3.17 ms, TR = 8.15 ms, flip angle = 9°, slice thickness = 1 mm, field of view [FOV] = 256 × 256 mm, matrix size = 256 × 256), DTI (TR = 7,300 ms, TE = 99 ms, flip angle = 90°, thickness = 3 mm, slices = 50, FOV = 256 mm × 256 mm, matrix size = 128 × 128, two averages, voxel size = 2 mm × 2 mm × 3 mm). DTI scan (b = 1,000 s/mm^2^) were acquired with 30 diffusion gradient orientations and the b = 0 repeated two times. The presence of nonhemorrhagic and micro‐hemorrhagic lesions was independently determined by experienced clinical neuroradiologists (with 9 and 10 years' experience) who assessed multiple modalities of neuroimaging data acquired at baseline (T1‐flair; T2‐flair; susceptibility weighted imaging, SWI).

### 2.5 Quality control

Head motion induces bias in DTI scalar measurements (Jenkinson, Bannister, Brady, & Smith, 2002; Ling, Merideth, et al., 2012). Quality assurances were conducted on head motions for all subjects and subjects were excluded from further analysis if they were identified as motion outliers (three standard deviations [*SD*] greater than their cohorts). The rotation and translation parameters from each DTI acquisition were obtained using FSL' s linear registration tool FLIRT of each brain volume to the averaged b0 volume (Mukherjee, Chung, Berman, Hess, & Henry, 2008). Of 184 subjects, none of them were discarded due to excessive motions. There was also no significant difference in the measurement of head motion between patients and controls for both cohorts (*p* > .5).

Quantitative estimation of signal‐to‐noise (SNR) value is challenging for DTI because of the different signal properties of the b0 and diffusion weighted images. Typically the b0 images are generally used and reflect the SNR measurements for several possibilities (Mukherjee et al., 2008). In this study, we used the motion corrected, co‐registered and averaged b0 volume output for each subject (Jenkinson et al., 2002). The mean SNR value (%, ± *SD*) of b = 0 s/mm^2^ images for all regions in vivo measurements was 37.2 ± 5.1% for original sample (34.3 ± 7.3% for replicated sample). Our data obtained good SNR (at least 20) to derive relatively reliable FA values according to (Mukherjee et al., 2008). There were also no significant difference in the measurement of SNR between patients and controls for these two cohorts (all for *p* > .4).

### 2.6 Calculation of imaging features

For DTI analysis, fractional anisotropy (FA) were generated using the Tract‐Based Spatial Statistics (TBSS) in the FMRIB Software Library (Smith et al., 2006). Image analysis using TBSS included the following steps: (a) nonlinear alignment of all subjects' FA images into a common space using the FMRIB nonlinear registration tool; (b) affine‐transformation of the aligned images into standard MNI152 1 mm space; (c) averaging of the aligned FA images to create a 4D mean FA image; (d) thinning of the mean FA image to create a mean FA “skeleton” that represents the centers of all white matter tracts common to the group; and (e) thresholding of the FA skeleton at fractional anisotropy≥0.2 to suppress areas of extremely low mean FA.

We then captured the lesion‐load diffusion abnormalities, separately summing clusters of abnormally high and low anisotropy, regardless of specific location. Specifically, the mean and SD of FA was first calculated for each voxel from the spatially normalized (whole‐brain fractional anisotropy template) sample of matched healthy controls (EZ‐MAP) (Lipton et al., 2012). The EZ used a bootstrap procedure to overcome the potential for sample‐to‐sample variation of reference healthy control. Control group, with an even distribution of age, gender and educational attainment that fully cover the range of the patients, was subdivided into two similar subgroups of controls each (“reference group” and “normal control subjects”). A linear regression model was also created to adjust the potential covariate effects of age, gender and educational attainment from the reference group (Hakulinen et al., 2012). The derived regression coefficients were used to FA images of another subgroup of control subjects (“normal control subjects”) and patients with mild TBI, but restricted to the locations where effects were significant (*p* < .05). The abnormal voxel was determined for patients and normal control groups separately based on the two criteria: (a) each voxel met the threshold *EZ* ∣  > 1.96 and is masked with the fiber tract defined by the Johns Hopkins University WM atlas and within the FA skeleton; (b) search for contiguous clusters meeting a size threshold (5%, corrected for multiple comparison) based on the Gaussian Random Field (GRF) theory (Friston, Worsley, Frackowiak, Mazziotta, & Evans, 1994). These thresholds are determined by the maximal discrimination between patients and normal controls based on ROC curve measured by a range of thresholds (Lipton et al., 2012). We then extracted abnormally high or low diffusion respectively for each fiber tract. Finally, 26 fiber tracts and 43 clusters (for either high or low diffusion) met the criteria. Then the mean value of FA for each cluster was used as the predictor feature from each mild TBI patient.

Besides, we also defined additional thalamo‐cortical tracts using probabilistic tractography as the supplement because the JHU atlas under‐represents subcortical and interhemispheric connections. Tractography was performed using the thalamus as the seed and the anterior cingulate gyrus, inferior frontal gyrus, and superior frontal gyrus as target regions. The projected tracts were then averaged across an independent cohort of 10 control subjects. We also repeated the EZ analysis and finally extracted 6 fibers and 10 clusters (for either high or low diffusion) met the criteria. Then the mean value of FA for each cluster was used as predictor feature. Overall, for each participant in this study, there were 53 imaging cluster features.

### 2.7 Imaging feature selection

These 53 imaging FA features were then entered into the feature selection procedures. Feature selection techniques have been widely adopted in brain analysis studies, in order to produce a small number of features for efficient classification or regression, and to reduce overfitting and increase the generalization performance of the model (Dosenbach et al., 2010; Drysdale et al., 2017). We adopted recursive feature elimination (RFE) procedure (Fagerholm, Hellyer, Scott, Leech, & Sharp, 2015), where features contributing little would be recursively eliminated until the optimal pattern that gave maximal performance was obtained. Every feature was ranked according to the weight vector (i.e., the importance in the final prediction) by using a linear kernel. The least important feature (in terms of its weight vector) was removed and a new SVM trained with the remaining features. The number of features used for the classifier was determined by the optimal accuracy of the classification performance.

### 2.8 Prognostic modeling and internal validation

**FIGURE 1:**
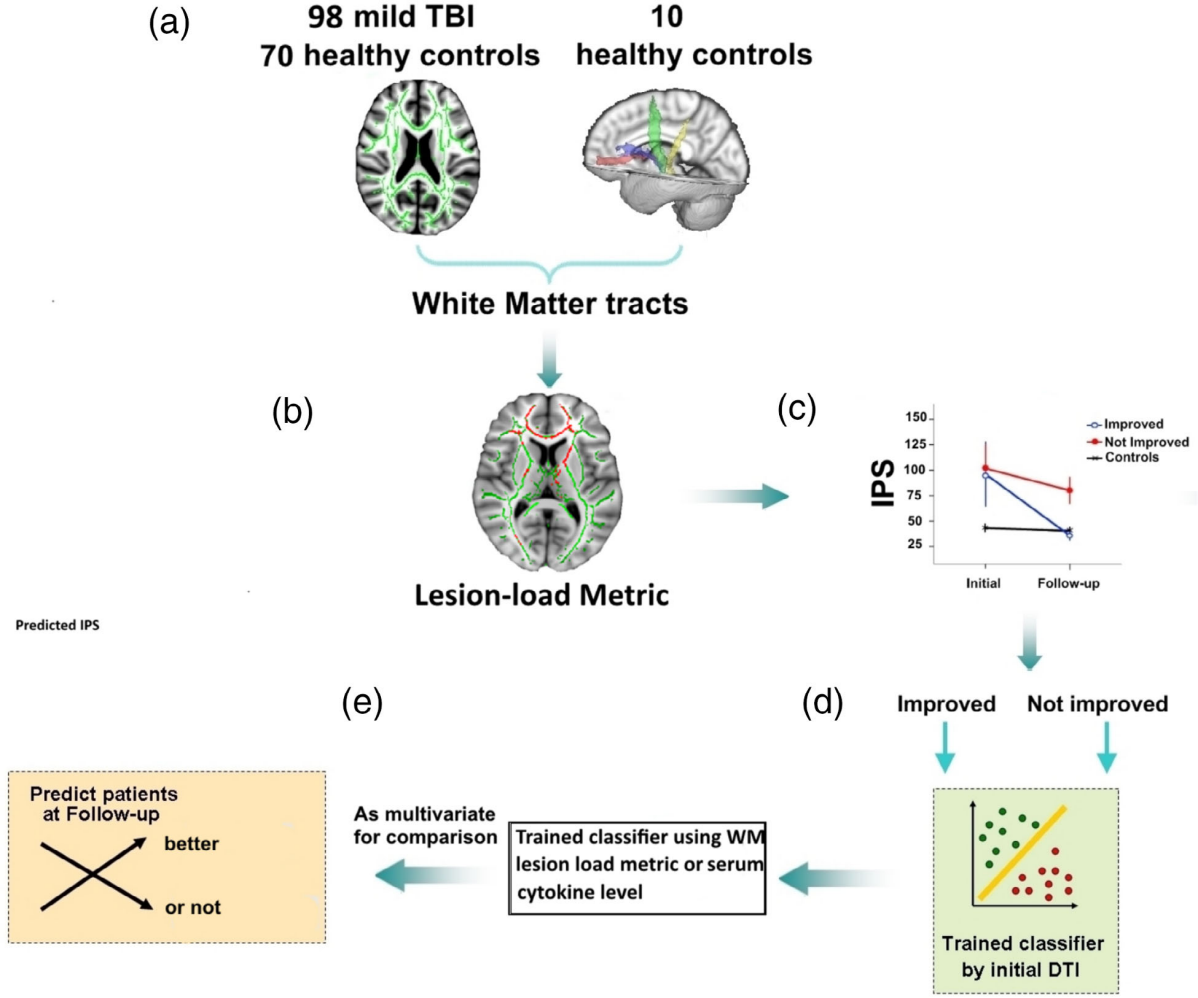
Summary of methods. (a) Skeletonized diffusion metric for white matter tracts was measured from 98 patients with mild TBI and 70 matched healthy controls. Additional thalamo‐cortical tracts were defined by using probabilistic tractography in 10 separate healthy controls. (b) lesion‐load analytical strategy to capture spatially heterogeneous white matter injuries from the skeletonized diffusion metric; (c) Patients were grouped into those whose information processing speed (IPS) score was improved to the normal level or not at follow‐up visit. (d) SVM was used to examine whether structural DTI measured at acute phase can divide patients into the above two groups. (e) Comparison of predicted performance with only lesion‐load abnormality features or combination with serum cytokine

For the original sample, support vector machines (SVM) (the LIBSVM classification library, http://www.csie.ntu.edu.tw/~cjlin/libsvm/) used the selected imaging features to examine whether acute features can divide patients into two groups (improved as class label +1 or not as −1at follow‐up) (Figure 1). For the internal validation of model, leave‐one‐subject‐out cross validation (LOOCV) was used to estimate classification (SVM) and prediction (SVR) accuracies (Dosenbach et al., 2010). LOOCV is a commonly implemented cross‐validation tool because it allows using most of the data for training and provides a conservative estimate of a classifier's or predictor's true accuracy (Kohavi, 1995). To avoid high degrees of variance produced by LOOCV, we used repeated subsampling validation to randomly select n subjects as the test sample and thus set aside whereas all remaining subjects formed the training sample (Cawley, 2006). The statistical significance of all LOOCV results was determined by using permutation testing by randomly reassigning class labels for 5,000 times (*p* < .05) for each n from 1 to 8. Using this approach we estimated the empirical cumulative distribution of the classifier and predictor accuracies under the null hypothesis (no discriminability) (Taylor & Noble, 2003).

Support vector regression (SVR) was used to predict continuous labels of neuropsychological scores in libSVM classification library (Vapnik, 2000). The model trained on diffusion metric to identify patient with IPS deficit was then adopted to predict IPS. We also tested whether this model can predict working memory profile, considering mediation effect of IPS observed in healthy and neurological changes in working memory (Fry & Hale, 2000; Kochunov et al., 2017).

### 2.9 Comparison of the single‐domain and combination models

Using the same modeling and validation method as described above, we examined predictability in the original dataset based on the serum cytokine features alone, or the combination of the two‐domain features.

### 2.10 External validation of model

External validation is essential to support the general applicability of a prediction model. We ensured external validity by testing the model in the replicate sample dataset, which included samples not involved in the development of the model. Using this prognostic model, all the determined optimal features and their weight vectors (i.e., the importance in the final prediction) were adopted in the replicated sample to divide patients into two groups (improved as class label +1 or not as −1at follow‐up). The performance of the classification, including the accuracy, sensitivity and specificity, was determined.

### 2.11 Statistical analysis

The Shapiro–Wilk W test was used to test for normality distribution of all continuous variables. The independent two‐sample *t* test and Mann–Whitney test were used to compare group differences based on data normality, respectively. Chi square analyses were applied to assess categorical variables. Effect sizes (Cohen's *d*) were computed to demonstrate the magnitude of observed differences. 95% confidence intervals (CI) were reported to convey the effects of sampling variation on the precision of estimated statistics (Chavalarias, Wallach, Li, & Ioannidis, 2016).

## 3 RESULTS

FLAIR and SWI images of all the subjects were reviewed by two board‐certified neuroradiologists to rule out nonhemorrhagic and micro‐hemorrhagic lesions. Fourteen patients and two healthy controls were excluded from the analyses due to gross abnormalities in the central cerebral white matter, visible on FLAIR or SWI images. Some subjects had small superficial hemosiderin staining but were not excluded, as these lesions are not expected to affect the central white matter tracts that this study aimed to analyze. Finally, a total of 98 patients and 70 HC met the inclusion criteria. Demographics, behavioral statistics and serum cytokines for original and replicated cohort of patients and controls were summarized in Table 1. There were no significant differences between healthy controls from the original and replication samples on major demographic variables (*p* > .1). Patients presented impaired performance on the IPS and working memory, compared with healthy controls in both original and replicated patient sample (all for *p* < .005). Causes for injury included motor vehicle accident (70% and 57% for original and replicated samples respectively), assaults (13% and 25% for original and replicated samples respectively), and fall (17% and 18% for original and replicated samples respectively). None of patients were with visible contusion lesions using conventional neuroimaging techniques and exhibited cerebral microbleeds on susceptibility weighted imaging. Serum levels of CCL2, IL‐1β, and IL‐6 in acute phase were higher in mTBI patients than in controls after Bonferroni correction (all for *p* < .001).

Longitudinal analyses were conducted to examine change in the IPS as a function of recovery. However, patient's performance on IPS and working memory did not recovery to normal level at follow‐up compared with healthy control (all for *p* < .005, Table 1).

### 3.1 DTI selected features

**FIGURE 2:**
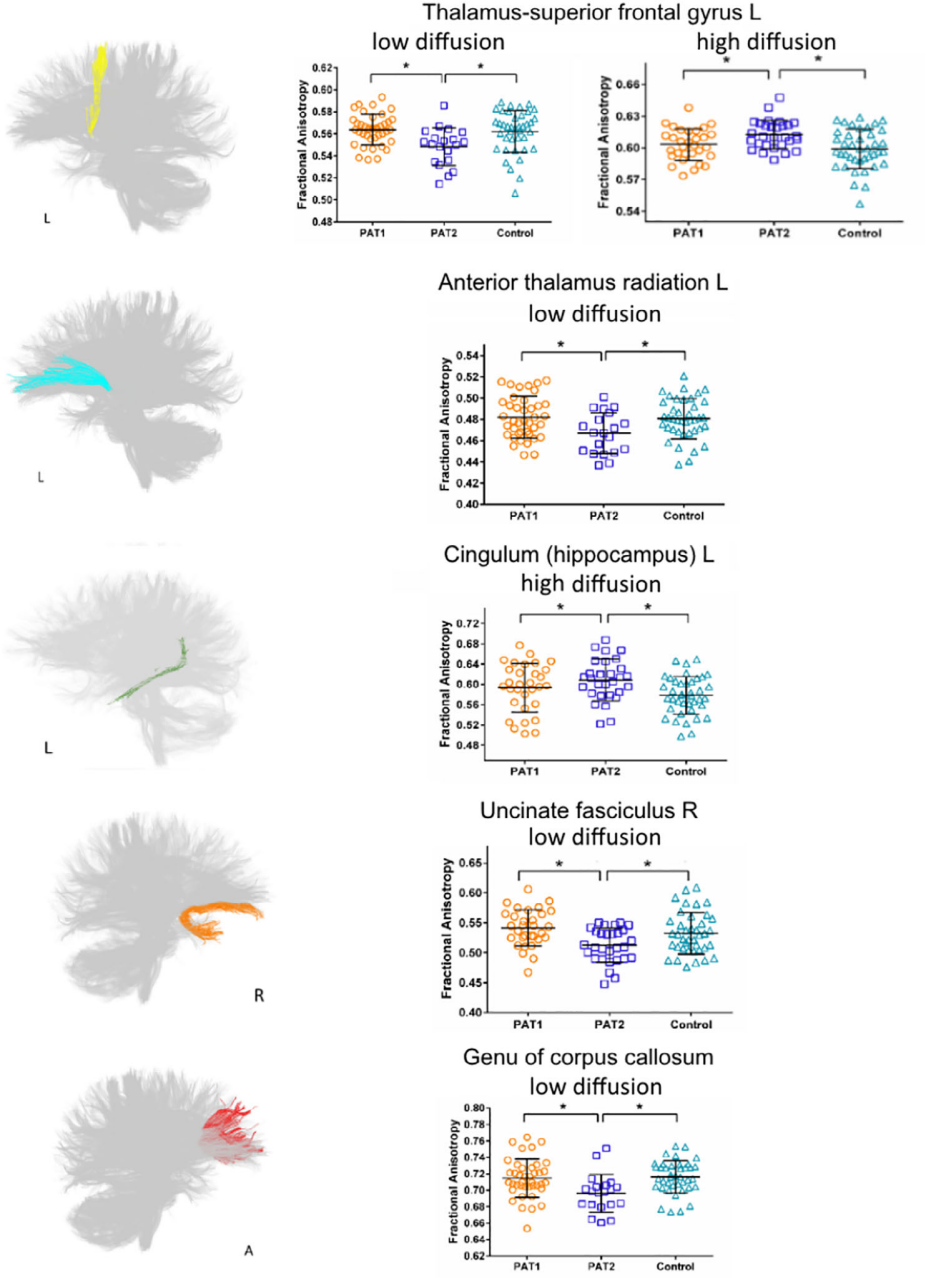
Most important weighted lesion‐load cluster in specific fiber tracts as predictors and showing significant differences between PAT1 and PAT2, as well as PAT2 and healthy controls (*p* < .05, Bonferroni correction for multiple comparisons). These tracts included the left thalamus‐SFG tract with both low and high diffusion clusters, left cingulum (hippocampus) with high diffusion cluster, left anterior thalamic radiation, right uncinate fasciculus and genu of CC with low diffusion cluster. PAT1, patients with recovery to the normal level for the IPS; PAT2, patients with incomplete recovery for the IPS; HC, healthy controls. IPS, information processing speed; L, left; R, right

Table 2 showed the final selected imaging features. Based on the lesion‐load detection method, we found both low and high anisotropic diffusion clusters for some fibers used as the predictors. These fibers included the bilateral thalamus‐superior frontal gyrus (SFG) tract, left corticospinal tract, right cingulum (hippocampus), right inferior longitudinal fasciculus (ILF) and right uncinated fasciculus (UF). Some tracts present only high diffusion clusters, such as the bilateral thalamus‐anterior cingulate tract, left cingulum (hippocampus), forceps minor, left ILF, right superior longitudinal fasciculus (SLF), left UF, body and splenium of corpus callosum (CC). Other tracts primarily exhibited low diffusion clusters, including bilateral anterior thalamic radiation (ATR), left cingulum (cingulate gyrus), right inferior fronto‐occipital fasciculus (IFOF), right SLF (temporal part) and genu of CC. Most important weighted lesion‐load clusters were then chosen based on: *i)* clusters with weight vector ranked as top 25% (6 for total 27 fibers); *ii)* presenting significant differences in patients without complete recovery compared with both patients with complete recovery and controls after Bonferroni correction for multiple comparisons, yielding an adjusted level of *p* < .008 ([0.05÷6] for 6 clusters). The left thalamus‐SFG tract with both low and high diffusion clusters, left cingulum (hippocampus) with high diffusion cluster, left anterior thalamic radiation, right uncinate fasciculus and genu of CC with low diffusion cluster became the most important predictors (Figure 2).

**TABLE 2:** The final selected white matter fibers (with clusters for either high or low diffusion) used as predictors and their normalized contribution weights (w)

White matter fibers	Weights for high diffusion	Weights for low diffusion
Thalamus‐anterior cingulate L	0.75	NS
Thalamus‐anterior cingulate R	NS	0.77
Thalamus‐inferior frontal gyrus R	NS	0.22
Thalamus‐superior frontal gyrus L	0.82	1
Thalamus‐superior frontal gyrus R	0.43	0.76
Anterior thalamic radiation L	NS	0.93
Anterior thalamic radiation R	NS	0.50
Corticospinal tract L	0.37	0.61
Cingulum (cingulate gyrus) L	NS	0.45
Cingulum (hippocampus) L	0.87	NS
Cingulum (hippocampus) R	0.81	0.46
Forceps minor	0.42	NS
Inferior fronto‐occipital fasciculus R	0.47	0.40
Inferior longitudinal fasciculus L	0.59	NS
Superior longitudinal fasciculus R	0.60	NS
Uncinate fasciculus L	0.44	NS
Uncinate fasciculus R	0.57	0.86
Superior longitudinal fasciculus (temporal) R	NS	0.78
Genu of corpus callosum	NS	0.83
Body of corpus callosum	0.45	NS
Splenium of corpus callosum	0.65	NS

Abbreviation: NS, not selected.

### 3.2 Predicting long‐term IPS deficits and internal validation

For the original sample, patients with incomplete recovery in IPS accounted for 33% of the whole cohort at follow‐up stage. SVM trained on the diffusion metrics at acute stage was firstly trained to discriminate mild TBI patients with and without IPS deficits at follow‐up. Multivariate fiber measures discriminated these subgroups of patients with high accuracy (92.1%; 95% CI, 91.6%–92.6%), sensitivity (99%; 95% CI, 99.1%–99.5%) and specificity (84.9%; 95% CI, 83.9%–85.9%) for classifiers trained on FA, which was significantly better than chance (*p* < .001).

### 3.3 Predicting neuropsychological functions in individual patient

**FIGURE 3:**
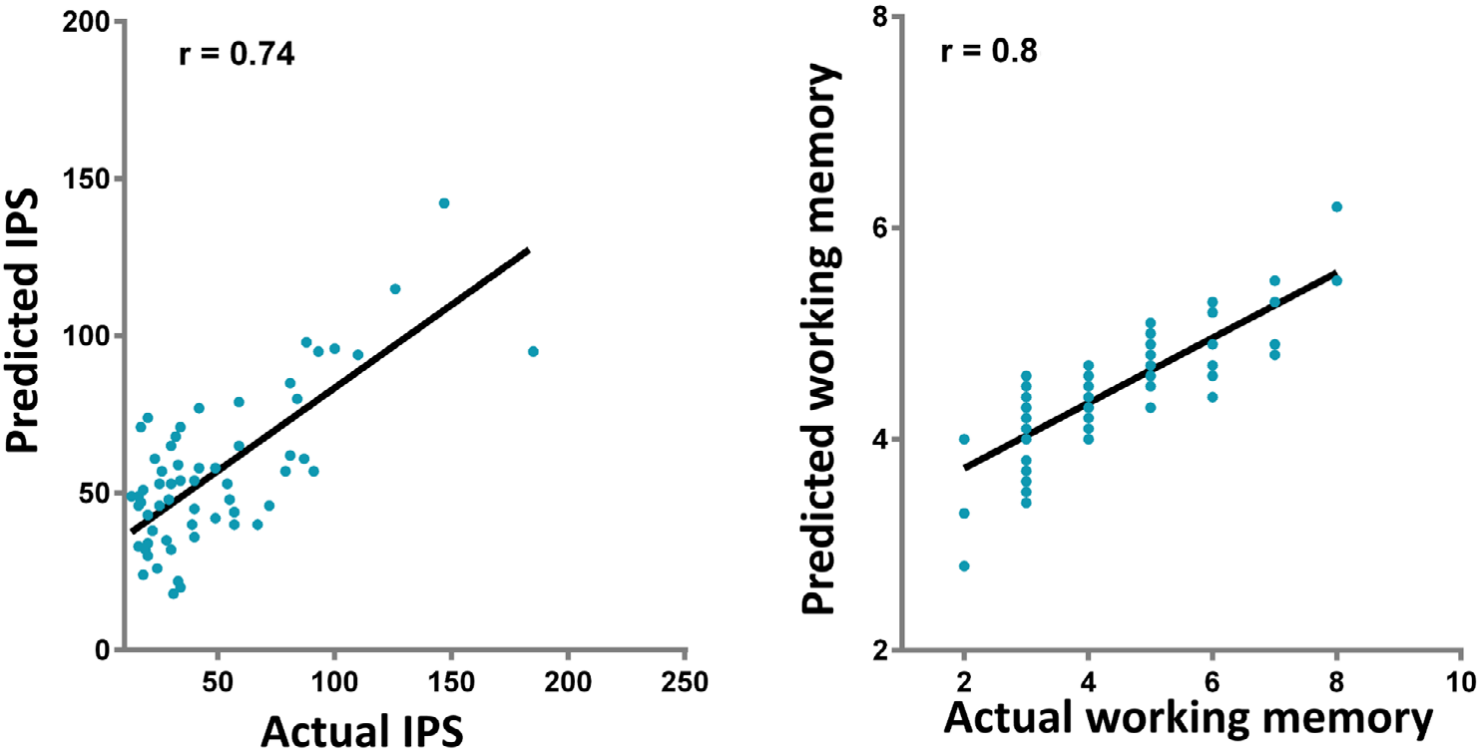
Cognitive function predicted using support vector regression (SVR). SVR was trained by using DTI classifier in identify patients with information processing speed (IPS) (rated by Trail‐making A test score, TMTA) deficit to predict the individual IPS profile (a) and working memory (b) (rated by back forward digit sequencing, BDS). There were significant positive relations between the true neuropsychological score and the predicted value for both IPS and working memory

We also trained SVM for regression using diffusion FA to predict the IPS scores at follow‐up. Spearman's correlation coefficient between actual IPS and predicted IPS was *r* = .74 (95% CI, 0.58 to 0.86; *p* < .001) (Figure 3a). The model trained on diffusion metric to identify IPS deficits was then adopted to predict individual working memory profile at follow‐up. Spearman's correlation coefficient between actual working memory and predicted working memory was *r* = .80 (95% CI, 0.67 to 0.92; *p* < .001) (Figure 3b).

### 3.4 Comparison of the single‐domain and combination models

The single serum cytokine features (assessed at acute phase) predicted this outcome on its own with 54% accuracy (*p* > .1). Combination of the FA metric and serum cytokine features further improved the prediction performance with high accuracy (96.7%; 95% CI, 96.4%–97.1%), sensitivity (99.5%; 95% CI, 99.3%–99.7%) and specificity (94.0%; 95% CI, 93.4%–94.7%).

### 3.5 Model external validation

The prediction model tested on the replicate sample also produced better performance using the diffusion features as predictors, with high accuracy (82.4%; 95% CI, 81.7%–83.1%), sensitivity (90.7%; 95% CI, 89.9%–91.5%) and specificity (74.1%; 95% CI, 72.9%–75.4%). Combination of the FA metric and serum cytokine features further improved the prediction performance with high accuracy (85.6%; 95% CI, 84.9%–86.3%), sensitivity (86.6%; 95% CI, 85.6%–87.5%) and specificity (84.6%; 95% CI, 83.6%–85.6%).

## 4 DISCUSSION

Persist core cognitive impairment following mild TBI represented the early brain pathology (i.e., white matter tract injury) and specific regional fiber tracts involvement. The power of lesion‐load diffusion measures to identify patients developing IPS deficits (92.1% accuracy) enhanced its possibility in diagnose for early prognostication. Combination of both diffusion metric and serum cytokine can further improve this accuracy (96.7%). Importantly, its clinical diagnosing potential was further verified by an independent replicate sample and its successful predictions for clinical outcomes (IPS and working memory). To the best of our knowledge, this is the first study using both the DTI metric and inflammation levels at the very early acute stage to identify the core cognitive deficits in chronic mild TBI. These findings provide targets for early interventions to improve outcome in risky patients with incomplete cognitive recovery after mild TBI, and warrant validation.

The study of risky patients with mild TBI is challenging because, in the absence of positive signs on conventional neuroimaging, one cannot be certain whether or when a given individual will develop ongoing cognitive sequelae. Our study identified early regional diffusion values can predict patients with core cognitive deficit (IPS) 6–12 month postinjury. This was consistent with one study that acute altered FA represents a clear neurobiological link with one‐year postinjury cognitive dysfunction after mild/moderate TBI (Croall et al., 2014). Discriminative fibers were mainly located in the thalamo‐cortical circuits and commissural pathways projecting or connecting the frontal regions, including the anterior thalamic radiation, forceps minor and genu of corpus callosum. The anterior thalamic radiation carries fibers from the brainstem and connects thalamus, striatum and anterior cingulate cortex to the anterior frontal region, which is involved in the IPS and planning complex behaviors (Floresco & Grace, 2003). The forceps minor is a part of the largest commissural fiber pathway connecting bilateral anterior frontal regions between two hemispheres (Fabri, Pierpaoli, Barbaresi, & Polonara, 2014). FA values in the forceps minor can be used in machine learning to predict cognitive impairments (Haller et al., 2010). This finding suggested that fiber pathways connecting the anterior and ventromedial nuclei of thalamus to the prefrontal cortex have tissue properties that enable better information flow across brain regions.

Our results presented the bidirectional fractional anisotropy changes and increased FA in the most of subcortical–cortical tracts and association fibers during the acute phase of mild TBI. By contrast, low fractional anisotropy changes were spatially distinct from regions of bidirectional and high fractional anisotropy. TBI‐induced FA increases have been reported clinically and in experimental blast TBI (Harris et al., 2016; Johnstone et al., 2015; Mayer, Hanlon, & Ling, 2015; Sidaros et al., 2008). FA increase was also presented in the widespread subcortical fibers at 1 week postinjury that persisted at 4 weeks after rodent controlled cortical impact injury (Harris et al., 2016). It was suggested as spontaneous axon sprouting occurs and lower brain structures may well attempt to regenerate after injury (Sidaros et al., 2008). Other study from histopathologic investigation has indicated that astrogliosis may also cause acute FA increases in mild TBI (Budde, Janes, Gold, Turtzo, & Frank, 2011). While, the genu of the CC exhibited low fractional anisotropy changes and consistent with previous study (Ling, Pena, et al., 2012). The functional significance of these changes remain unclear, but these findings may indicate that white matter impairments in mild TBI was regional tract–specific.

Our current study also indicated that serum cytokine levels are increased after mild TBI and persist from acute to chronic phase (Sun et al., 2019). Cytokine levels in acute phase can predict the patients' IPS at 3 month postinjury. In the present study, combination of the DTI metric and serum cytokine features can further improve the identification accuracy of patients with cognitive IPS deficits at 6–12 month follow‐up. Systemic inflammation can trigger neuroinflammation through circumventricular organs, vagal afferents, or the brain endothelium (Miller, Maletic, & Raison, 2009), undermining the microstructural integrity of white matter (Arfanakis et al., 2013; Briones & Woods, 2014), disrupting microglia function in synaptic plasticity and reducing cognitive functioning.63 These results, in conjunction with other studies, indicated that enhancing the white matter tract integrity and anti‐inflammation treatment showed the potential in the improvement of cognitive functioning following mild TBI.

Several limitations of this study have to be mentioned, including the restricted generalizability of results. First, the study was primarily specific to the recruited cohort. Though cross‐validation procedures enhance the generalization of predicted model, further study is needed to adequate sampling not only of a population as a whole but also of the diversity of individuals. Second, future work with larger patient groups could incorporate this type of technique, and should explicitly model a large number of clinical variables that are known to be important in explaining outcome, such as MR spectroscopic, MRI volumetric, preinjury factors, neurobehavioral and genetic markers (Tremblay, Iturria‐Medina, Mateos‐Perez, Evans, & De Beaumont, 2017).

## 5 CONCLUSION

For clinical practice, this early identification could be done at discharge from the emergency department, measuring the white matter integrity and blood serum cytokine. With this approach, the selection of those patients who have to be seen at the outpatient clinic can be narrowed down. Several studies investigating outcomes after mild TBI found that indices of injury severity (CT‐abnormalities, Glasgow Coma Score, age and education levels) found to be reliable predictors for incomplete recovery (Cnossen et al., 2017; Greenberg et al., 2017). However, our study further proved that early diffusion measures within specific tracts and inflammation levels can be effective in predicting subtypes of patients with incomplete recovery in cognitive domain. This model was also verified by an independent replicate sample which further enhanced its clinical potentials. Future validation study should be worthwhile to validate by large samples to investigate the specific cutoffs from DTI metrics and serum cytokine.

## CONFLICT OF INTEREST

The authors declare no competing financial interests.

## Data Availability

De‐identified data are available from the corresponding author upon reasonable request subject to a material transfer agreement.
